# SARS-CoV-2 Seroprevalence among Health Care Workers—A Voluntary Screening Study in a Regional Medical Center in Southern Germany

**DOI:** 10.3390/ijerph18083910

**Published:** 2021-04-08

**Authors:** Katharina Müller, Philipp Girl, Michaela Ruhnke, Mareike Spranger, Klaus Kaier, Heiner von Buttlar, Gerhard Dobler, Johannes P. Borde

**Affiliations:** 1 Bundeswehr Institute of Microbiology, 80937 Munich, Germany; katharina5mueller@bundeswehr.org (K.M.); heinervonbuttlar@bundeswehr.org (H.v.B.); gerharddobler@bundeswehr.org (G.D.); 2 German Centre for Infection Research (DZIF), Partner Site Munich, 80937 Munich, Germany; 3 Praxis Dr. J. Borde, Gesundheitszentrum Oberkirch, 77704 Oberkirch, Germany; michaela.ruhnke@gmail.com (M.R.); mareike.spranger@yahoo.de (M.S.); johannes.borde@web.de (J.P.B.); 4 Institute of Medical Biometry and Statistics, Faculty of Medicine and Medical Center, University of Freiburg, 79098 Freiburg, Germany; kaier@imbi.uni-freiburg.de; 5 Department of Medicine II, Division of Infectious Diseases, Faculty of Medicine and Medical Center, University of Freiburg, 79098 Freiburg, Germany

**Keywords:** COVID-19, SARS-CoV-2, health care personnel, seroprevalence

## Abstract

Severe acute respiratory syndrome coronavirus-2 (SARS-CoV-2) is associated with a potentially severe clinical manifestation, coronavirus disease 2019 (COVID-19), and currently poses a worldwide challenge. Health care workers (HCWs) are at the forefront of any health care system and thus especially at risk for SARS-CoV-2 infection due to their potentially frequent and close contact with patients suffering from COVID-19. Serum samples from 198 HCWs with direct patient contact of a regional medical center and several outpatient facilities were collected during the early phase of the pandemic (April 2020) and tested for SARS-CoV-2-specific antibodies. Commercially available IgA- and IgG-specific ELISAs were used as screening technique, followed by an in-house neutralization assay for confirmation. Neutralizing SARS-CoV-2-specific antibodies were detected in seven of 198 (3.5%) tested HCWs. There was no significant difference in seroprevalence between the regional medical center (3.4%) and the outpatient institution (5%). The overall seroprevalence of neutralizing SARS-CoV-2-specific antibodies in HCWs in both a large regional medical center and a small outpatient institution was low (3.5%) at the beginning of April 2020. The findings may indicate that the timely implemented preventive measures (strict hygiene protocols, personal protective equipment) were effective to protect from transmission of an airborne virus when only limited information on the pathogen was available.

## 1. Introduction

Severe acute respiratory syndrome coronavirus-2 (SARS-CoV-2) first appeared at the end of 2019 in Wuhan, China. The viral pathogen was rapidly identified and characterized by virus culture as well as whole genome sequencing [1], and first data on the epidemiological dynamics of transmission were obtained [2]. The disease was later named coronavirus disease 2019 (COVID-19). Symptoms can range from mild flu-like symptoms [3] to severe systemic (multiple organ dysfunction) [4] and pulmonary disease with fatal complications [5], especially in risk groups like the elderly. Initial observations from China reported that 13.8% of all cases suffered a severe course of the disease [6], and 6.1% took a critical course. Despite promptly imposed infection control measures, SARS-CoV-2 spread around the world. The virus is mainly transmitted from person to person by droplet infection via infectious aerosols but it can also remain viable on different surfaces for hours and even days [7]. The World Health Organization (WHO) officially announced an outbreak of pandemic scale on 11 March 2020. Soon after, SARS-CoV-2 infections became a notifiable disease in Germany, and the German public health authorities, led by the Robert Koch Institute (RKI), reported a total of 174,355 confirmed cases and 7914 casualties related to SARS-CoV-2 on 17 May 2020. The diagnostic procedure of an acute infection is based on direct virus detection in oro- or nasopharyngeal swabs via RT-qPCR [8]. Past infections on the other hand can be assessed using SARS-CoV-2-specific serological testing such as ELISA, neutralization assays (NT), or immunofluorescence assays [9,10]. IgG seroconversion was reported to be very similar to that in SARS-CoV infections and occurred 7 (50% seroconversion rate) to 14 days (100% seroconversion rate) post symptom onset. Interestingly, as described for SARS and Middle East respiratory syndrome (MERS), IgM seroconversion was not significantly earlier than IgG [8]. Less data are available on SARS-CoV-2-specific IgA antibodies, with one study reporting a median time for IgA seroconversion of 11 days (range: 5–20) [11].

Health care workers (HCWs) are the frontline workforce of every health care system and thus particularly at risk to acquire a SARS-CoV-2 infection while caring for COVID-19 patients. Therefore, various institutions like the WHO (World Health Organization), CDC (Centers for Disease Control and Prevention), RKI (Robert Koch-Institut), and medical societies issued safety recommendations for HCWs to take personal protective measures especially during high-risk procedures such as endotracheal intubation or bronchoscopy [12]. At the same time, infected HCWs also pose a risk to other patients, colleagues, and their own families, especially when the infection is asymptomatic.

Studies from different countries and regions report very different seroprevalence rates among health care workers. A study in Sweden, for example, found a seroprevalence of almost 19% among HCWs at the beginning of the pandemic (April–May 2020), which was significantly higher than the seroprevalence of the general population at that time, which was reported at 7.3% for Stockholm [13]. Similarly, high prevalence was found in a study in New York City, which found a seroprevalence of almost 14% among public health workers. Interestingly, however, the seroprevalence among the general population was about the same at the time of the study, so that no increased risk could be assumed for HCWs [14]. In contrast, there are also studies that report rather low seroprevalence among health care employees. In Denmark, for example, seroprevalence at the start of the pandemic (April 2020) was just under 4% among health care workers and thus only slightly higher than in the control group [15]. A study conducted among health care employees of a multistate hospital network in the USA found a similarly low seroprevalence of 6% (April–June 2020), with considerable variations by locations. However, seroprevalence in HCWs generally correlated with community cumulative incidence, and thus no significantly higher risk of infection was found for HCWs [16]. Up to date, only little data are available on the seroprevalence of SARS-CoV-2-specific antibodies in HCWs in Germany at the beginning of the pandemic [17]. However, this kind of information is important for potential future outbreaks. It can help to evaluate the usefulness of different general guidelines, instructions, etc., established in medical facilities at the beginning of a pandemic, when only limited information on the pathogen might be available.

The aim of this study was to (I) get insights into the intra-hospital seroepidemiology of SARS-CoV-2 among HCW at the beginning of the pandemic (April 2020), (II) determine whether medical personnel were at greater risk of infection at that time, and (III) whether the risk of infection was different for personnel in a hospital compared to personnel in a regional medical center. For this purpose, we analyzed serum samples of 198 volunteer HCW (all involved in direct patient care) taken as part of occupational health screenings for the presence of SARS-CoV-2-specific antibodies using a commercially available ELISA. Additionally, we established an in-house NT to confirm ELISA findings and to determine the neutralizing capabilities of the detected antibodies. Interestingly, the overall seroprevalence was low, and we detected neither significantly higher antibody levels among HCW compared to the general population nor any difference between hospital staff and other medical staff in small outpatient facilities.

## 2. Materials and Methods

### 2.1. Setting

The county Ortenaukreis is part of the federal state of Baden-Wuerttemberg in Southern Germany. It has 429,479 inhabitants (December 2018) and directly neighbors the French Department Grand-Est with its capital Strasbourg. The counties administrative capital is the city of Offenburg (59,646 inhabitants), where the regional 630-bed medical center is located. It provides multidisciplinary, specialized, and advanced medical services, including a level-1 center for traumatology, pulmonology and thoracic surgery, obstetrics and neonatal care, visceral surgery, urology, and a certified cancer center. It is divided into two separate facilities both located within the city, one major (461 beds) and one minor (171 beds) facility. The medical center is an affiliated teaching hospital of the University Medical Center in Freiburg.

The first three cases of SARS-CoV-2 infections in the county were notified to the regional public health authorities on 3 March 2020. By 22 March 2020, the number of notified cases had already passed 100. Strict hospital infection control measures in the context of COVID-19 patients were imposed at the beginning of March 202,0 and detailed recommendations for the care and treatment of COVID-19 patients were issued based on the interim guidelines of the German Society of Hospital Hygiene published in January 2020. This included stricter regulations on hand disinfection as well as respiratory hygiene. In addition, the use of medical masks (at least, FFP-2 filter) and face shields was recommended when seeing patients with respiratory and flu-like symptoms, and the appropriate equipment was provided. Additionally, a strict visitation policy was issued at the end of March, and no more visitors were allowed into the hospital to further reduce the likelihood of virus introduction. At the same time, all HCWs were ordered to wear surgical masks at all times in addition to the previously established recommendations. In contrast, the outpatient sector initially suffered from a shortage of personal protective equipment until May 2020, as most of the material available at this time was given to the inpatient sector. The outpatient sector was advised by public health authorities to wear surgical masks (if available), implement strict hand hygiene protocols, and keep sufficient distance from patients and colleagues whenever possible. At the same, patients were publicly advised to cancel or reschedule all non-essential appointments and, when experiencing flu-like symptoms, to first contact their respective primary care physician or the responsible health authorities by telephone to coordinate further action.

### 2.2. Samples

Individual SARS-CoV-2 serological diagnostics was offered on seven consecutive days—2–8 April 2020—exclusively to HCWs at the major hospital facility of the regional medical center. Additionally, the same testing was also offered to HCWs of several small outpatient facilities (i.e., medical personnel from a general practitioner’s office, an ophthalmology practice, a pediatric practice, and one pharmacy). Both the hospital and the outpatient facilities reported that they had seen and treated patients with laboratory-confirmed COVID-19. HCWs were defined as those involved in direct patient care (i.e., physicians, nurses, and supporting medical personnel), leading to a total number of 707 HCWs in the regional medical center and an additional 40 in the outpatient facility. As this study was performed at an early stage of the pandemic, no further specific inclusion or exclusion criteria were applied other than the strict requirement for direct and regular patient contact, thus excluding administrative staff among others.

Every participant filled in a short anamnestic questionnaire at the time of the blood sampling (Figure S1). The survey included demographic questions as well as questions regarding previous symptoms (cough, fever, myalgia, anosmia), the need for hospital admission (intensive care unit (ICU), non-ICU setting, need for oxygen or ventilator support), and potential results of previous SARS-CoV-2 specific tests (e.g., RT-qPCR results of nasopharyngeal swabs) (S1 File). Serum samples were sent to the Bundeswehr Institute of Microbiology in Munich for further analyses. Samples were processed strictly anonymized within the central diagnostic unit, and all results were reported to the participant by an attending physician. All tests were performed with the anonymized serum samples, which were stored at −80 °C.

### 2.3. Case Definition

COVID-19 is a notifiable disease in Germany. National case definitions were issued by the RKI (www.rki.de (accessed on 30 March 2020)). Case definitions are based on the direct detection of SARS-CoV-2 either by RT-PCR or culture.

### 2.4. SARS-CoV-2 ELISA

Anti-SARS-CoV-2 IgG and IgA ELISAs were performed according to the manufacturer’s instructions (Euroimmun, Lübeck, Germany), and ratios were calculated correspondingly. Samples were evaluated as either not elevated (ratio < 0.8), indeterminate (0.8 ≤ ratio ≤ 1.1), or elevated (ratio > 1.1) for both IgA and IgG, as suggested by the manufacturer. Both elevated and indeterminate results in either one of the ELISAs were interpreted as “reactive”, and all sera classified as such were subsequently analyzed using an in-house neutralization assay.

### 2.5. In-House Neutralization Assay/Tissue Culture Infectious Dose 50 Test (TCID50)

An in-house neutralization assay was established as a TCID50 assay according to a previously described standard procedure [18] to check for SARS-CoV-2-specific antibodies with neutralizing capabilities. SARS-CoV-2 (strain MUC IMB-1) was grown in Vero E6 cells (ATCC CRL-1586), and virus stocks (40–60 TCID/50 µL) were prepared and stored at −80 °C until further use. Patient sera (duplicates) were diluted (1:5) in Minimal Essential Medium (MEM, plus MEM Non-Essential Amino Acids Solution plus Antibiotic–Antimycotic Solution; all Invitrogen, ThermoFisher Scientific, Darmstadt, Germany) in a 96-well cell culture plate (Greiner bio-one, Frickenhausen, Germany). One positive and one negative serum were used as controls along with a mock control and a virus re-titration on every plate. The virus was added to each well, and the serum–virus solution was incubated for one hour at 37 °C (5% CO_2_). Afterwards, Vero E6 cells (1 × 10^4^ cells/50µL) were added to each well and incubated for another 72 h at 37 °C (5% CO_2_). The supernatants were then discarded, and the 96-well plates were fixed in 13% formalin/PBS and stained with crystal violet according to a previously described protocol [19].

### 2.6. Statistical Methods

Analyzes were exploratory and performed without adjustment for multiple testing. Between-group differences were analyzed using Fisher’s Exact Test.

## 3. Results

### 3.1. Statistical Methods

Overall, 200 serum samples were obtained from HCWs. Two serum sample were excluded due to missing/inconclusive data (*n* = 198); 178 sera were collected from personnel of the regional medical center, whereas 20 sera came from personnel of the outpatient facilities. Regarding the medical center, approximately 25% (178/707) of all HCWs involved in direct patient care were tested. In contrast, 50% (20/40) of all HCWs in the outpatient facility involved in direct patient care were tested. The mean age of the HCWs from the medical center was 41 years, 34% (61/178) were men, and 63% (113/178) women—in four (4/178) cases, no gender was documented. The mean age of the HCWs from the outpatient institution was 51 years, 10% (2/20) were men, and 90% (18/20) women. In total, 26% (52/198) of the tested HCWs reported symptoms prior to being tested (Table 1).

### 3.2. History of SARS-CoV-2 Swabs Results

Overall, 14 participants (14/198) had reportedly been tested for SARS-CoV-2 via oro-nasopharyngeal swabs and RT-qPCR prior to serum sampling. Of these, 12 reported being tested for corona-specific symptoms, while 2 were tested for other reasons. In addition, 4/14 reported positive swab results, whereas 10 reported negative results. RT-qPCR diagnostics was provided by various certified commercial laboratories in the region using previously published standard protocols. Swabs were taken between 13 and 22 days (mean 17.3 days) prior to serum sampling.

### 3.3. SARS-CoV-2 ELISA

**Figure 1:**
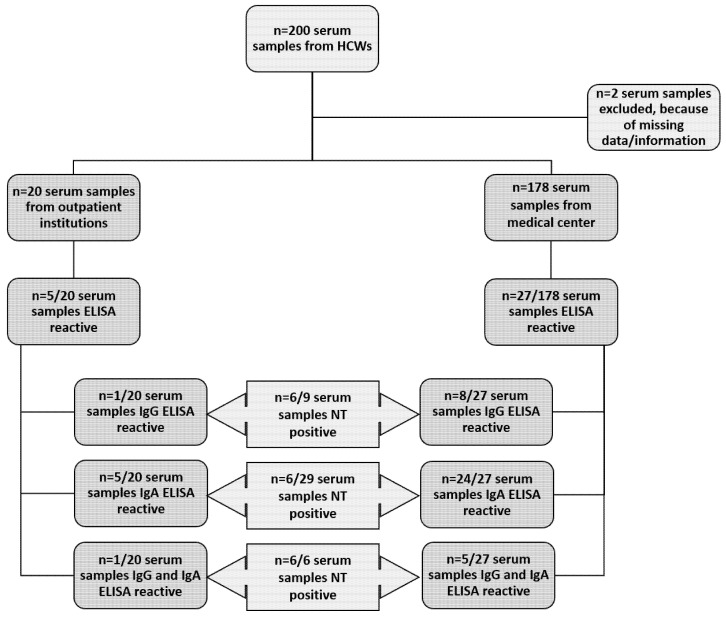
Detailed overview of the serum samples. In total, 198 samples from health care workers (HCWs) were screened using commercially available anti-SARS-CoV-2 ELISAs (IgA and IgG). ELISA-reactive sera were further analyzed using an in-house neutralization assay (NT).

All 198 samples were tested using both IgG- and IgA-specific ELISA (Figure 1). Reactive results were seen in 16% (32/198) of ELISA tests, of which 5% (9/198) were reactive for SARS-CoV-2 IgG antibodies, 15% (29/198) were reactive for SARS-CoV-2 IgA antibodies, and 3% (6/198) were reactive for both. Among the 178 samples from the regional medical center, 15% (27/178) showed reactive ELISA results, 5% (8/178) were reactive for IgG, 13% (24/178) were reactive for IgA, and 3% (5/178) were reactive for both. HCWs from the outpatient institution showed reactive ELISA results in 25% (5/20) of the tested sera, of which 5% (1/20) were reactive for IgG, 25% (5/20) were reactive for IgA, and 5% (1/20) were reactive for both. Of the 32 patients with reactive ELISA results, 14 (44%) reported symptoms prior to testing. Conversely, only 25% (13/52) of patients who had previously reported symptoms showed reactive ELISA results (Table 1).

### 3.4. In-House Neutralization Assay/Tissue Culture Infectious Dose 50 (TCID50)

**Figure 2:**
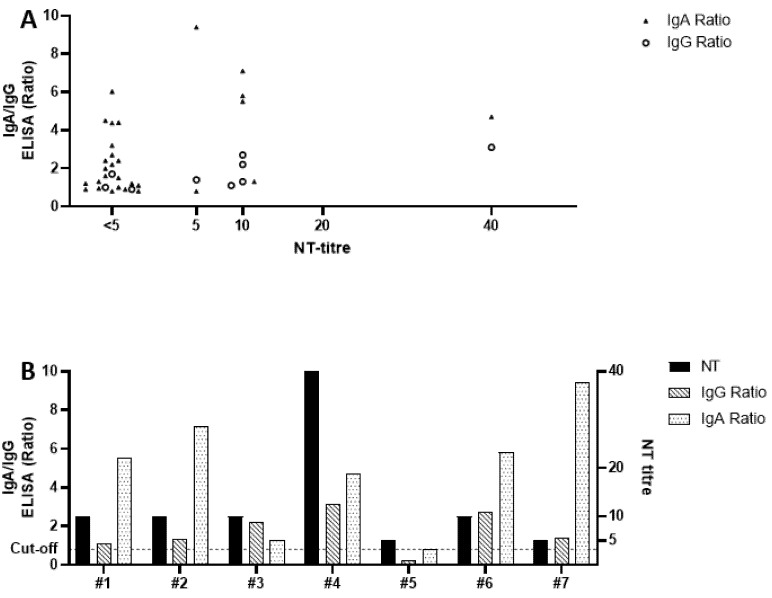
Comparison of ELISA ratios and neutralization assay (NT) titers. (**A**). A total of 32 samples were reactive in either one or both ELISA. The majority (23/29) of IgA-reactive samples showed no neutralizing effect, whereas two-thirds (6/9) of IgG-reactive samples were also positive for SARS-CoV-2-neutralizing antibodies. (**B**). Of the seven samples (#1–7) with detectable levels of neutralizing antibodies, all were reactive for IgA, and all but one (#5) were reactive for IgG. Overall, when comparing ELISA results and NT titers, no prediction could be made about the levels of neutralizing antibodies from the ELISA ratios (neither IgA nor IgG).

All sera that were reactive in either one or both ELISAs (*n* = 32) were further analyzed using our in-house NT. Overall, neutralization (NT titer ≥ 5) could be detected in seven sera (22%) (Figure 2B), including all four sera (100%) from participants who reported previous positive swab results. Of the nine samples that were reactive for IgG, six (67%) showed neutralizing antibodies, whereas three sera (33%) showed no neutralizing effect (Figure 2A). The mean ratio of IgG-reactive sera with confirmed neutralization was 2.0, while sera with no detectable neutralization had a mean ratio of 1.2. Of the 29 samples with a reactive result in the IgA ELISA, only seven (24%) showed neutralizing antibodies, and the mean ELISA ratio of these sera was 6.4. The remaining 23 IgA-reactive samples (79%, mean ratio 2.2) were unable to neutralize the virus (Figure 2A). Interestingly, all six samples that were reactive for both IgA and IgG were capable to neutralize SARS-CoV-2, while only one sample with confirmed neutralization was only reactive for IgA (Figure 2B #5). All seven patients (100%) with NT-confirmed antibodies also reported symptoms.

## 4. Discussion

To get further insights into the seroepidemiology of SARS-CoV-2 in HCWs, we offered voluntary SARS-CoV-2 antibody testing to medical personnel in a regional medical center and additionally to HCWs in selected outpatient facilities. We used commercially available SARS-CoV-2-specific IgA and IgG ELISA for screening and used an in-house neutralization assay to confirm ELISA-reactive results.

Overall, 16% of the tested samples were reactive for either IgA or IgG or for both, indicating the presence of SARS-CoV-2-specific antibodies and thus a possible past infection with SARS-CoV-2. The findings from the regional medical center showed seroconversion in 15% (27/178) of the tested HCWs, whereas 25% (5/20) of HCWs of the outpatient facilities were reactive in at least one ELISA. Of those reactive, the number of IgA-reactive samples from the medical center was significantly higher (*p* < 0.001) compared to samples reactive for IgG (i.e., 89% vs. 30%). Among all reactive samples from the outpatient facilities, the number of IgA-reactive samples was 100% and thus significantly higher (*p* < 0.05) than the number of samples reactive for IgG (20%). One explanation might be that IgA serves as the first line of defense and is among the first type of immunoglobulins to be seen during an infection [17]. For SARS-CoV-2, it was described that IgA antibodies are detectable as early as five days post symptom onset, thus those samples might be from people who had just recently been infected with SARS-CoV-2 and had not yet experienced IgG seroconversion. However, the same study also found, indeed, a high sensitivity but lower specificity (73%) for the IgA ELISA that was used in this study, suggesting that a certain amount of IgA-reactive samples might be false positive. This would be in line with the fact that only 24% of IgA-reactive samples could be confirmed by NT [11]. Samples solely reactive for IgG can be explained by the fact that while IgG seroconversion is slower, it is largely responsible for long-term immunity and thus persists in the circulation for an extended period of time, while IgA levels decrease much faster after an infection [20].

Epidemiological data indicate that there is immunity after COVID-19, at least to some extent [21]. However, it is difficult to derive any definitive statements regarding (long-term) immunity solely based on ELISA, given its limitations in sensitivity and specificity as previously explained. We therefore performed an in-house neutralization assay, which nears the biological equivalent of protection against infection in vitro, on every sample with elevated or indeterminate ELISA results. The fact that only 22% of the reactive sera neutralized SARS-CoV-2 in cell culture is surprising, yet in line with a very recently published report that not all SARS-CoV-2-specific antibodies have neutralizing capabilities, especially not those produced at the beginning of infection, where mostly IgA would be present [22]. This also supports our finding that most ELISA-reactive sera that could be confirmed by NT were either IgG-reactive (67%) or reactive for both IgG and IgA (100%), while only 24% of IgA-reactive samples showed neutralizing antibodies. In accordance to the serological results of the “Heinsberg Study”, we could also see that sera with a low IgG ELISA ratio tended to show no neutralizing capabilities [23].

Based on our combined ELISA and NT results, we conclude that in our study population, 3.5% of participating HCWs showed a specific and confirmed antibody response indicating a present or past infection with SARS-CoV-2. Among the personnel of the outpatient facilities, only one person (5%) showed seroconversion and confirmed neutralizing antibodies, which is in line with the fact that this person reported a positive SARS-CoV-2 RT-qPCR result prior to sampling. Interestingly, this person also reported close contact with a confirmed SARS-CoV-2-infected individual outside of her workplace, making it very likely that this participant did not get infected during work, theoretically reducing the work-related seroprevalence to 0% in the outpatient facilities. As for the medical center, 3.4% of the participating HCWs showed SARS-CoV-2-specific neutralizing antibodies, and one of them reported a PCR-confirmed infection. Additionally, the same person also reported a possible infection during a stay in a high-risk area, theoretically reducing the seroprevalence due to work-acquired SARS-CoV-2 to 2.8%. The other five participants stated that while they did not knowingly have contact with SARS-CoV-2-infected people outside of work, they could not rule it out with certainty either. Interestingly, all of them also reported symptoms, indicating that at least none of the NT-confirmed seropositive participants were silent or asymptomatic carriers.

Taken together, our data suggests that the overall seroprevalence of SARS-CoV-2-specific antibodies is low in HCWs, at least in this region of Southern Germany and at the time of this study. No significant difference (*p* = 0.58) in seroprevalence could be detected when comparing the results of the medical center with the results of the outpatient institution. This may be somewhat surprising, given the difference in personal protective equipment that was available to the hospital staff and the staff at the outpatient settings. One possible explanation could be the difference in patient clientele. While all participating outpatient facilities confirmed that they also came into contact with SARS-CoV-2-positive patients, their patient load and the extent of patient contact is not comparable to that of a hospital. Therefore, while the protective equipment was less prevalent, the same was true for patient contact and thus for the risk of infection while working.

In addition, our results underline the importance of gathering background information when examining seroprevalence in a population to avoid overestimation. Given the evidently high contagiousness [24] of SARS-CoV-2, the overall low seroprevalence might indicate abidance to implemented hygiene protocols, their effectiveness, and the appropriate use of personal protective measures; however, said abidance was not examined in this study. At the same time, it must be kept in mind that this specific region is currently not and has not been a SARS-CoV-2 hot spot, thus limiting the chance of contact to some extent.

In general, our results, though based on a limited number of samples, are in line with a recently published study from a referral tertiary university medical center in Germany caring for severe COVID-19 patients, which detected the presence of SARS-CoV-2-specific antibodies in only 1.6% (5/316) of HCWs [17]. Newspaper articles and comments from another regional medical center in Hesse, which were published in different national media covering the pandemic, also indicate a very low seroprevalence of SARS-CoV-2 antibodies of only 1% (18/1800) among tested HCWs [25]. There are, however, also reports of high seroprevalence among HCWs in Germany. A recently published study reported a 10- to 20-fold increased risk for HCWs to acquire a SARS-CoV-2 infection at work [26]. However, this study was done in a large academic teaching hospital with a COVID-19-positive patient load of up to 34.5%, which is not comparable to the number of COVID-19 patients seen in the medical center investigated in this study (36 cases between 1 January and 7 April 2020).

Our study, of course, has some limitations. We examined a non-structured population of HCWs, since participation was voluntary, and no specific inclusion or exclusion criteria were applied. This could lead to an overestimation of seroprevalence, as individuals who feel like they might have had a higher chance of infection may be more likely to participate. However, we tried to take some precautions to reduce the volunteer bias as much as possible. For example, we used the possibility for the participants to receive their own test results as an incentive to increase the overall likelihood of participation. In addition, by making direct patient contact a requirement for participation, we tried to make our study population as consistent as possible and reduce the likelihood that some participants would have significantly more incentive to participate than others (for example, because of significant differences in exposure risk). At the same time, of course, the timing of this study is another limitation worth noting. The sampling took place in April, relatively early in the pandemic. However, seroprevalence is dynamic, and because serum samples were screened for antibodies, our data represent the prevalence of prior infections but not acute infections. Later conducted studies will thus yield different results. Several other studies [13,14,15] have also been conducted at very similar time points in different countries. The early timing in particular may allow conclusions to be drawn in order to derive guidelines that can be useful in future outbreaks to protect medical personnel especially during the early phase of a pandemic, when only little is known about the pathogen.

Another problem is, of course, that there is no way of telling if a positively tested HCW got infected during medical work. However, we were able (through personal communication) to identify two participants with NT-confirmed antibodies who most likely acquired SARS-CoV-2 outside of work. We provide, however, early seroepidemiological data for HCWs in a region of Germany with moderate SARS-CoV-2 incidence. It would be of great interest to collect follow-up samples, especially of all participants who were ELISA-reactive but had no neutralizing antibodies. This would give insight into whether these ELISA results might have been truly false or if it simply takes some additional time after seroconversion for neutralizing antibodies to be produced, as suggested by Seydoux et al. [22].

## 5. Conclusions

In conclusion, the overall seroprevalence of SARS-CoV-2 antibodies in the tested HCWs was low at the beginning of April 2020. It did not significantly differ between the medical center and the outpatient institution and did not exceed 5%. The data indicate that basic infection-control measures employed during the early phase of the pandemic in hospitals and outpatient institutions are effective and help to prevent nosocomial patient-to-staff spreading, even when other countermeasures like vaccines are not available.

## Figures and Tables

**Table 1:** Overview of the symptoms reported by the participants. Overall, 26.3% (52/198) of participants reported symptoms prior to sampling; 25% of participants who reported symptoms (13/52) showed reactive ELISA results. SARS-CoV-2, severe acute respiratory syndrome coronavirus-2.

Symptoms	Number of Participants Reporting Symptoms Prior to SARS-CoV-2 ELISA Testing (*n* = 52)	Number of Participants Reporting Symptoms with Reactive ELISA Results (*n* = 13)
**Dyspnea**	8/52 (15%)	4/13 (31%)
**Cough**	40/52 (77%)	11/13 (85%)
**Fever**	17/52 (33%)	5/13 (38%)
**Myalgia**	18/52 (35%)	7/13 (54%)
**Anosmia**	9/52 (17%)	7/13 (54%)
**Hospitalization required**	1/52 (2%)	1/13 (8%)

## Data Availability

The data presented in this study are available on request from the corresponding author. The data are not publicly available due to privacy restrictions.

## Supplementary Materials

The following are available online at https://www.mdpi.com/article/10.3390/ijerph18083910/s1, Figure S1: Anamnestic Questionnaire (translated and original version). Every participant was required to fill in this short anamnestic questionnaire prior to blood sampling.
