# Supplementary material for: SARS-CoV-2 Seroprevalence among Health Care Workers—A Voluntary Screening Study in a Regional Medical Center in Southern Germany
Source: Int J Environ Res Public Health. 2021 Apr 8;18(8):3910. doi: 10.3390/ijerph18083910 (PMC8068211; doi:10.3390/ijerph18083910)
Supplement: Supplementary file 1 [file ijerph-18-03910-s001.pdf]

SARS-CoV-2 serological diagnostics

Background: Currently, the healthcare system at all levels of care is heavily burdened with the direct and indirect consequences of the Sars-CoV-2 pandemic. Medical personnel, along with appropriate personal protective equipment, are a critical resource during this time.

SARS-CoV-2 seroconversion is a strong indication of having been infected with SARS-CoV-2 in the past and, according to current knowledge, could confer potential immunity and reduce the risk of re-infection, at least for a certain amount of time. The test procedure offered here is a serological assay (non-accredited procedure) to determine your SARS-CoV-2 serum status (i.e. the presence or absence of antibodies against SARS-CoV-2). Current data suggests that seroconversion usually occurs 14-21 days after infection. Individual results and their interpretation will be discussed with you by your attending physician. Participation is completely voluntary. The declaration of consent can be revoked at any time and your test results will be deleted. Non-participation is not associated with any disadvantages.

Surname:	
First name:	
Date of birth:	
Phone / email	
Medical facility (address)	
Ward/unit	

☐ I have previously been tested **positive** for Sars-CoV-2 by throat swab / PCR on:
___ . ___ . _______
I experienced symptoms: **yes** □ no □
If yes (multiple selection):
□ cough □ fever □ shortness of breath □ pain in the limbs □ loss of sense of smell/taste
□ I had to be hospitalized (24h or more)
□ normal ward
□ intensive care unit
□ non-invasive ventilation □ invasive ventilation

☐ I have previously been tested **negative** for Sars-CoV-2 by throat swab / PCR on:
___ . ___ . _______
I experienced symptoms: **yes** □ no □
If yes (multiple selection):
□ cough □ fever □ shortness of breath □ pain in the limbs □ loss of sense of smell/taste
□ I had to be hospitalized (24h or more)
□ normal ward
□ intensive care unit
□ non-invasive ventilation □ invasive ventilation

☐ I have **not** been tested for Sars-CoV-2 by throat swab / PCR.
I experienced symptoms: **yes** □ no □
If yes (multiple selection):
□ cough □ fever □ shortness of breath □ pain in the limbs □ loss of sense of smell/taste
□ I had to be hospitalized (24h or more)
□ normal ward
□ intensive care unit
□ non-invasive ventilation □ invasive ventilation

Please contact us if you have any questions:

- PD Dr. J.P. Borde (johannes.borde@web.de)
- Lt. Colonel (MC) Prof. Dr. G. Dobler (gerharddobler@bundeswehr.org)

☐ **Yes, I consent** to the collection of a blood sample. My blood sample will sent to the Bundeswehr Institute of Microbiology of the German Armed Forces (Neuherbergstraße 11, 80937 Munich, Head of Institute: Colonel (MC) PD Dr. R. Wölfel) for SARS-CoV-2 specific serological diagnostics.

Signature serum donor: ______________________________ date: __ . __ . ______
